# Identification and functional prediction of long non-coding RNAs related to oxidative stress in the jejunum of piglets

**DOI:** 10.5713/ab.23.0202

**Published:** 2023-08-25

**Authors:** Jinbao Li, Jianmin Zhang, Xinlin Jin, Shiyin Li, Yingbin Du, Yongqing Zeng, Jin Wang, Wei Chen

**Affiliations:** 1 Key Laboratory of Efficient Utilization of Non-grain Feed Resources (Co-construction by Ministry and Province), Ministry of Agriculture and Rural Affairs, Shandong Provincial Key Laboratory of Animal Biotechnology and Disease Control and Prevention, College of Animal Science and Technology, Shandong Agricultural University, Tai’an City, 271018, China

**Keywords:** Jejunum, lncRNA, Oxidative Stress, Piglet, Transcriptome

## Abstract

**Objective:**

Oxidative stress (OS) is a pathological process arising from the excessive production of free radicals in the body. It has the potential to alter animal gene expression and cause damage to the jejunum. However, there have been few reports of changes in the expression of long noncoding RNAs (lncRNAs) in the jejunum in piglets under OS. The purpose of this research was to examine how lncRNAs in piglet jejunum change under OS.

**Methods:**

The abdominal cavities of piglets were injected with diquat (DQ) to produce OS. Raw reads were downloaded from the SRA database. RNA-seq was utilized to study the expression of lncRNAs in piglets under OS. Additionally, six randomly selected lncRNAs were verified using quantitative real-time polymerase chain reaction (qRT–PCR) to examine the mechanism of oxidative damage.

**Results:**

A total of 79 lncRNAs were differentially expressed (DE) in the treatment group compared to the negative control group. The target genes of DE lncRNAs were enriched in gene ontology (GO) terms and Kyoto encyclopedia of genes and genomes (KEGG) signaling pathways. Chemical carcinogenesis-reactive oxygen species, the Foxo signaling pathway, colorectal cancer, and the AMPK signaling pathway were all linked to OS.

**Conclusion:**

Our results demonstrated that DQ-induced OS causes differential expression of lncRNAs, laying the groundwork for future research into the processes involved in the jejunum’s response to OS.

## INTRODUCTION

The term “oxidative stress” was first coined in 1985 [1]. Oxidative stress (OS) is defined as disruption of the prooxidant–antioxidant equilibrium, which leads to the production of reactive oxygen species (ROS) [2]. ROS are short-lived, strongly reactive oxygen-containing molecules that may harm DNA and change the DNA damage response [3]. ROS are crucial second messengers in many intracellular signaling cascades that try to keep the cell in equilibrium with its immediate surroundings. As ROS accumulate, OS occurs when the ROS level exceeds the maximum limit of the body’s antioxidant defence system [4]. The intestinal barriers and intestinal cells are destroyed under OS conditions, leading to severe inflammatory bowel disorders and even bowel cancer. In ulcerative colitis, immune cells such as T cells cross the epithelial barrier, releasing inflammatory mediators and worsening mucosal damage [5,6]. Numerous elements in animal production can cause OS and damage cellular antioxidant defences. Weaned piglets may experience growth retardation, sickness, or even mortality as a result of OS [7]. Oxidative stress can result in subpar animal health, a decline in animal production efficiency, and significant financial losses in animal husbandry systems [8].

Homeostasis is maintained by cells through transcription and posttranscriptional regulation, which results in variations in gene expression [9,10]. LncRNAs are transcripts that are more than 200 nucleotides (nt) in length and lack an efficient open reading frame for translation. These transcripts typically regulate mRNA expression levels [11], nuclear organization [12], and diverse developmental processes such as differentiation [13]. LncRNAs serve critical functions in the cellular response to OS [14]. LncRNA NEAT1 is associated with H_2_O_2_-induced oxidative damage, and melatonin can attenuate H_2_O_2_-induced oxidative damage through the upregulation of lncRNA NEAT2 [15]. The lncRNA H19 is dramatically downregulated in the cochleae of old mice. In H_2_O_2_-stimulated HEI-OC1 cells, overexpression of H19 reduces mitochondrial ROS production and the apoptosis ratio. The mechanism is that lncRNA H19 protects cochlear hair cells from OS via the miR-653-5p/SIRT1 axis [16]. However, few lncRNAs induced by OS in the jejunum of piglets have been reported.

Diquat (DQ) is a fast-acting herbicide used to suppress terrestrial and aquatic plants as a contact and preharvest desiccant. Its ability to produce reactive oxygen and nitrogen species contributes to its toxic potential [17]. In addition to serving as an important agricultural and economic animal, the domestic pig (*Sus scrofa*) can serve as a model organism for medical research. Domestic pigs also produce a significant amount of meat for human consumption. Therefore, it is necessary to clarify the changes in lncRNA expression in piglets under OS. The purpose of this research was to explore lncRNAs related to OS between DQ-treated piglets and negative control (NC) piglets.

## MATERIALS AND METHODS

### Ethics statement

The animal study was reviewed and approved by the Animal Ethics Committee of Shandong Agricultural University, China, and performed in accordance with the Committee’s guidelines and regulations (Approval No.: 2004006).

### Experimental design and data collection

The experimental design and data for this study were obtained from our previous research [18]. The experimental design was as follow: 12 male Landrace piglets weaned at 21 days were collected and divided into the OS group and the NC group. In the OS group, piglets were intraperitoneally injected with DQ (Sigma-Aldrich, Saint Louis, USA) at 10 mg/kg body weight, while the NC group received an equivalent volume of isotonic saline. The trial lasted for seven days. Three piglets were selected from both the OS group and the NC group. After slaughter, the jejunum tissues were collected. Raw data with SRA number PRJNA661634 were downloaded with sratoolkit (v3.0.0). Six samples (three from the OS group and three from the NC group) from this dataset were used for this study. Of these, L01, L02, and L03 were the NC samples, and L04, L05, and L06 were the OS samples.

### Quality control for raw reads

The raw reads were quality-controlled before read alignment. Fastp (v0.23.2) [19] was used to filter the raw reads further. The reads with adapters, reads with unknown sequences, and low-quality reads were removed for quality control.

### Read alignment, assembly and quantification

Sequencing reads were aligned using HISAT2 (v2.2.1) [20] against the reference genome (*Sus_scrofa.Sscrofa*11.1.106.chr.gtf). To predict lncRNAs, the reads were then assembled and merged using StringTie (v2.2.1) [21]. Transcript expression levels were calculated for each sample using FeatureCounts (v2.0.3) [22] to obtain the gene counts. To reflect the expression levels of transcripts more realistically, the fragments per kilobase of transcript per million fragments mapped (FPKM) values were used as measures of transcript expression as calculated by Rstudio (v4.1.3).

### Identification of lncRNA

The lncRNAs were screened according to their characteristics. Briefly, the merged transcripts were compared by GffCompare (v0.12.6) [23] with the *Sus scrofa* reference genome. i) Transcripts with class_code “i” (fully contained within a reference intron), “u” (intergenic), “x” (exonic overlap on the opposite strand), “o” (other same strand overlap with reference exons), “j” (multi-exon with at least one junction match) were selected. ii) Those transcripts that were more than 200 nucleotides in length and had an exon number greater than or equal to 2 were further considered for the identification of novel lncRNAs. iii) CPC2 (v3.0) [24] and CNCI (v2.0) [25] were used to identify whether the transcript is encoded. When the score <0, the transcript was considered incapable of encoding. The transcripts that were able to be compared to the Pfam [26] database were transcripts with a certain protein domain; they were considered to have coding ability, while the transcripts that were incomparable were considered potential lncRNAs. The criterion for Pfam domain screening was an E-value <1×10^−5^. Transcripts with an E-value >1×10^−5^ were screened and retained. iv) Those transcripts with FPKM >0.1 were retained for the identification of novel lncRNAs. The identification of novel lncRNAs was considered complete after the above screening. The merged transcripts of the six samples were annotated to the *Sus scrofa* genome to obtain known lncRNAs.

### Differential expression analysis

DESeq2 [27] (v1.34.0) was employed for performing the differential expression analysis based on the counts of genes in samples with biological replicates. Upregulated genes showed higher expression levels in the OS group compared to the NC group in the current study, whereas downregulated genes exhibited lower expression levels in the OS group compared to the NC group. Differentially expressed (DE) lncRNAs were defined as genes with a false discovery rate <0.05 and log2(fold change) ≥1.

### Target gene prediction of differentially expressed lncRNAs

Bedtools [28] (v2.30.0) was used to identify cis-target genes for DE lncRNAs. Neighbouring genes within 100,000 bp of DE lncRNAs were considered cis-target genes of the lncRNAs [29]. The Pearson correlation coefficient method [30] was used to predict trans-target genes. When the sample size was greater than or equal to six, the Pearson correlation coefficient method was used to analyse the correlation between lncRNAs and protein-coding genes among samples. Then, lncRNA–mRNA gene pairs with absolute correlation values (|cor|) >0.95 and p<0.05 were retained.

### Enrichment analysis of target genes

To further understand the function of DE lncRNAs, the gene ontology (GO) and Kyoto encyclopedia of genes and genomes (KEGG) databases were used for enrichment analysis of target genes of DE lncRNAs. The GO database contains three main categories of functional information: the biological processes, molecular function, and cellular component categories. Each of these categories contains a hierarchical network of terms that describe different aspects of gene function. KEGG provides pathway maps, gene annotations, and other related information to study the functions of genes within specific pathways. The target genes were then uploaded into the DAVID [31] database 2021. GO terms and KEGG pathways with p<0.05 were considered significant by the DAVID database. To obtain the circos plot, the DAVID results were submitted to SangerBox (3.0).

### Alternative splicing analysis

The pre-mRNAs transcribed from genes can undergo various splicing events, where different exons are selected to generate diverse mature mRNAs. These mature mRNAs are then translated into different proteins, contributing to the diversity of biological traits. ASprofile [32] was used to classify 12 types of alternative splicing (AS) events for each sample.

### Quantitative real-time polymerase chain reaction analysis

Six DE lncRNAs were randomly selected to verify whether their expression was consistent with the trends of the RNA-seq results. Total RNA was extracted from piglet jejunal tissues using TRIzol (Invitrogen, Carlsbad, CA, USA). An Agilent 2100 Bioanalyzer (Agilent Technologies, Santa Clara, CA, USA) was employed to assess RNA quality. The cDNA was then synthesized. For the relative quantification of lncRNA, glyceraldehyde-3-phosphate dehydrogenase (GAPDH) was employed as an internal reference gene. Primers of selected lncRNAs (Table 1) were designed and then synthesized by Accurate Biotechnology Co., Let, Changsha, China. The quantitative real-time polymerase chain reaction (qRT-PCR) system for lncRNA had a total volume of 20 μL. The system consisted of 10 μL of 2X SYBR Green Pro Taq HS Premix, 0.4 μL of forward primer, 0.4 μL of reverse primer, 2 μL of cDNA template, and 7.2 μL of RNase-free water. Run the formulated 20 μL system on a Light Cycler 96 real-time PCR system (Roche, Basel, Switzerland) with the following program: 95°C for 30 s followed by 40 cycles of 95°C for 5 s and 60°C for 30 s. The 2^−ΔΔCT^ relative quantification approach [33] was utilized to perform the quantitative analysis of the data.

## RESULTS

### Overview of the sequencing data

The GC content of each sample was greater than 50.28%, Q20% was above 97.71%, Q30% was above 93.50%, and N% was below 0.01% after quality control (Table 2). This indicated that the clean reads obtained were suitable for subsequent transcriptome analysis. In total, 95.33% to 96.67% of the clean reads of individual samples were mapped to the *Sus scrofa* reference genome. A total of 84.05% to 87.18% of the mapped reads were considered unique mapped reads, while only 4.96% to 7.18% of the mapped reads were multiple mapped reads (Table 3). The mapping results indicated that the quality and reliability of the sequencing data were relatively high.

### Filtering of lncRNAs

**Figure 1:**
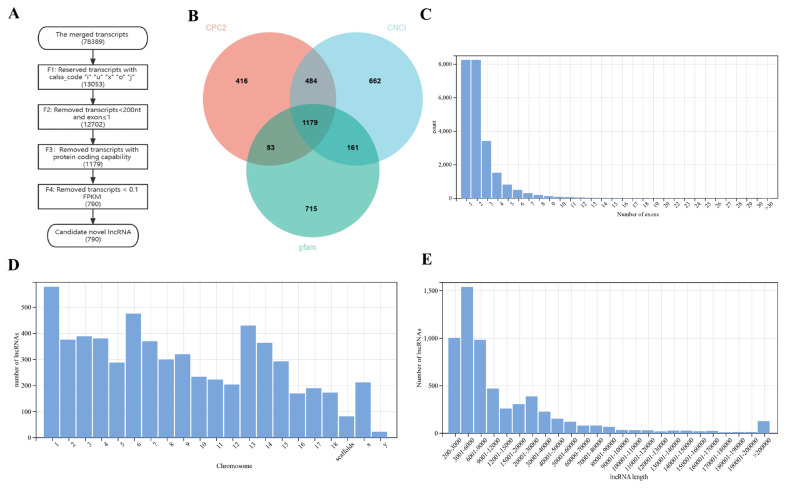
Screening of novel lncRNAs and genomic characterization of lncRNAs. (A) The flowchart of identifying novel lncRNA. (B) The Venn diagram of CPC2, CNCI and pfam software prediction. Pink represents CPC2, blue represents CNCI, green represents pfam. (C) Number of lncRNAs exons. (D) The chromosome distribution of lncRNAs. (E) Transcript length of lncRNAs.

Overall, 78,398 transcripts were used as input. A total of 790 candidate novel lncRNAs were finally identified (Figure 1A). A total of 13,053 transcripts were kept by filtering and retaining transcripts with class_codes “i”, “u”, “x”, “o”, “j”. A total of 83.35% of the initial transcripts were filtered; this was the step that filters out the most transcripts in identifying novel lncRNAs. After removing transcripts that were less than 200 nt in length and had an exon numbers less than 2, 12,702 transcripts remained. Only 0.48% of transcripts were further filtered out in this process. A total of 2,486 transcripts were retained through CNCI filtering; 2,162 transcripts were retained through CPC2 filtering; and 2,138 transcripts were retained through Pfam protein structural domain analysis. The results of the three software programs were intersected, and 1,179 transcripts were preserved (Figure 1B). A total of 9.07% of transcripts were further filtered out in this process. Transcripts with low expression were then removed. A total of 0.49% of transcripts were further filtered out in this process. Finally, 790 transcripts were retained. The 790 transcripts obtained were considered to be novel lncRNAs. The merged transcripts were compared with the *Sus scrofa* reference genome, and the transcripts with low expression were removed, resulting in 5,289 known lncRNAs. Ultimately, a total of 6,079 lncRNAs were identified.

### Genomic characterization of lncRNAs

The exon number distribution statistics of lncRNAs showed that the highest number of exons was 1, 2 (Figure 1C). Most lncRNAs were distributed on chromosome 1 in jejunal tissue, followed by chromosome 6 and chromosome 13 (Figure 1D). The length distribution statistics of lncRNAs showed that the most common length bin of the lncRNAs in the samples was 3,001 nt to 6,000 nt, followed by 200 nt to 3,000 nt and 6,001 nt to 9,000 nt (Figure 1E).

### Identification of differentially expressed lncRNAs

**Figure 2:**
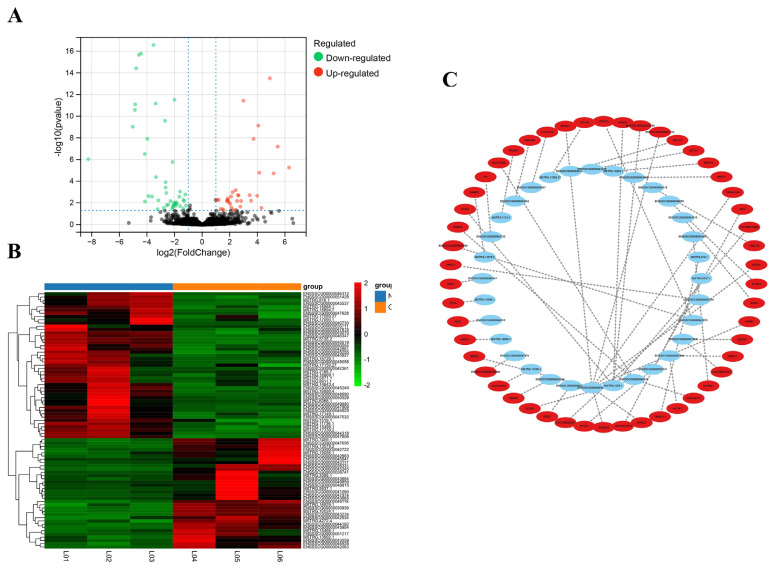
Statistics of DE lncRNAs and network. (A) Volcano plot analysis of 79 DE lncRNAs. The red dot represents the up-regulated gene, the green dot represents the down-regulated gene (B) Heatmap plots of DE lncRNAs. Rows represent lncRNAs, columns represent samples. The red color represents the higher expression of the gene in the sample and the green color represents the lower expression of the gene in the sample. (C) Interaction network for top 50 of lncRNA-mRNA gene pairs. The blue notes represent lncRNA. The red notes represent trans-target gene. DE, differentially expressed.

After differential expression analysis in the OS group and NC group, 79 DE lncRNAs (34 lncRNAs were upregulated and 45 lncRNAs were downregulated) were screened (Figure 2A; Supplementary Table S1). The heatmap showed that the expression levels of DE lncRNAs (Supplementary Table S2) were significantly different between the OS group and the NC group (Figure 2B).

### Target gene prediction of DE lncRNA

Of the 79 DE lncRNAs, 61 lncRNAs had cis-target genes within 100,000 bp upstream and downstream, in a total of 135 targets. A total of 12,980 lncRNA–mRNA pairs were predicted to be associated by the Pearson correlation coefficient method. The top 50 lncRNA-mRNA gene pairs (Supplementary Table S3) with the most significant p-values were shown in Figure 2(C). In this figure, the DE lncRNAs point towards their target mRNAs, representing the differential expression of lncRNAs that regulated their target genes in trans.

### Gene ontology enrichment analyses

**Figure 3:**
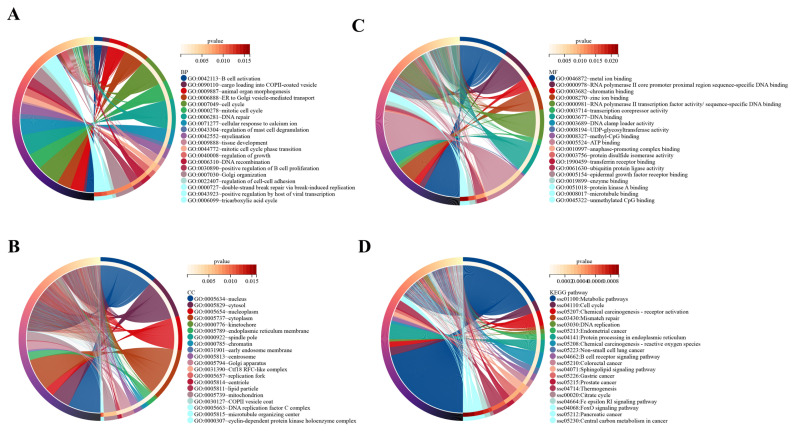
TOP 20 of GO enrichment analysis and KEGG pathways. In the circle diagram of GO enrichment and KEGG pathways, the circle is divided into a left half and a right half. In the left semicircle, the different colors represent different genes. In the outermost right semicircle, the different colors represent the different GO enrichments and KEGG pathways and the color of the inner side of the circle connected to the outermost side represents the p value, and the color changes from white to red as the p value increases. (A) Biological processes. (B) Cellular components. (C) Molecular functions. (D) KEGG pathways. GO, gene ontology; KEGG, Kyoto encyclopedia of genes and genomes.

In the present study, the target genes of DE lncRNAs were significantly enriched in 68 BP, 40 CC, and 48 MF categories (Supplementary Table S4). The top 20 BP terms mainly included B-cell activation, cargo loading into COPII-coated vesicle, cell cycle, DNA recombination, regulation of growth, tissue development, and other terms (Figure 3A). The top 20 CC terms mainly included nucleus, cytosol, nucleoplasm, cytoplasm, kinetochore, chromatin, and other terms (Figure 3B). The top 20 MF mainly included RNA polymerase II core promoter proximal region sequence-specific DNA binding, chromatin binding, methyl-CpG binding, protein kinase A binding, and other terms (Figure 3C).

### Kyoto encyclopedia of genes and genomes pathway analyses

A total of 4,645 target genes of DE lncRNAs were significantly enriched in 96 signaling pathways (Supplementary Table S5). The top 20 KEGG pathways mainly included metabolic pathways, cell cycle, chemical carcinogenesis-receptor activation, mismatch repair, DNA replication, chemical carcinogenesis -ROS, and other pathways (Figure 3D). Among the 96 KEGG pathways associated with OS were chemical carcinogenesis-ROS, colorectal cancer, Parkinson disease, insulin pathway, cellular senescence, Alzheimer’s disease, and other pathways.

### Alternative splicing analysis

**Figure 4:**
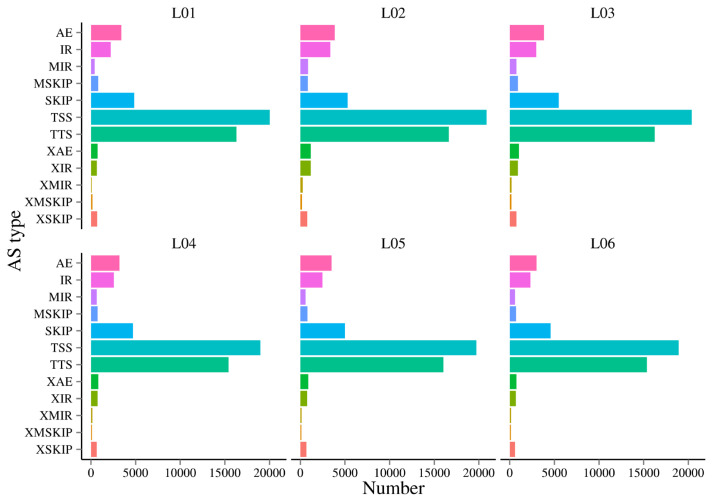
Statistics of AS events. The x-axis represents the quantity of AS events for each category. The y-axis represents the classification of AS events. Different colors show different types of AS events. AS, alternative splicing.

AS is an important mechanism of gene expression regulation, playing a crucial role in the normal functioning and disease development of organisms. In this research, a total of 12 AS events were detected (Figure 4). In this study, it was found alternative 5’ first exon (TSS) and alternative 3’ last exon (TTS) accounted for 67.84% to 72.13% of the AS events. This suggests that TSS and TTS were the two most prevalent AS events observed in this research.

### Quantitative real-time polymerase chain reaction validation of the gene expression data from RNA-seq

**Figure 5:**
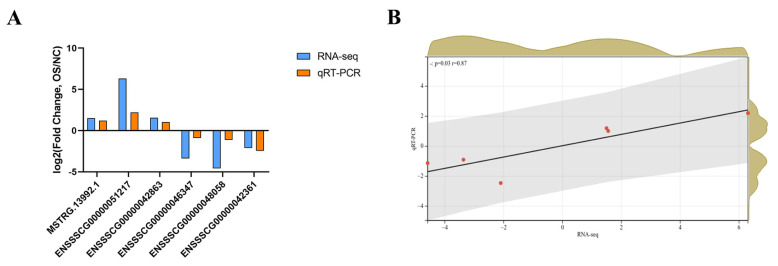
(A) Validation by qRT-PCR of 6 randomly selected DE lncRNAs from RNA-seq. (B) Correlation between RNA-seq and qRT-PCR. qRT-PCR, quantitative real-time polymerase chain reaction; DE, differentially expressed.

To validate the accuracy of RNA-seq, six DE lncRNAs (three upregulated lncRNAs and three downregulated lncRNAs) were randomly selected to perform qRT-PCR (Figure 5A). The correlation and p-value were obtained after linear fitting of the log_2_ (fold change). The results showed that there was a significant correlation between the RNA-seq and qRT-PCR (r = 0.87; p = 0.03 for DE lncRNAs) (Figure 5B). This also indicated the accuracy of high-throughput sequencing results.

## DISCUSSION

As a vital organ, the intestine not only takes in nutrients but also stops harmful substances such as bacteria and endotoxins from getting through the intestinal wall and into the body’s tissues, organs, and microcirculation [34]. Studies in the past have revealed that intraperitoneal injection of DQ can induce OS in piglets [35,36]. The DQ was thus used to construct a model of OS in piglets to study the effects of OS [37,38]. To further investigate the molecular mechanism of DQ-induced damage, RNA-Seq was used to identify DE lncRNAs. Overall, there were substantial variations in lncRNA expression in the jejunum between the OS and NC groups. Between the two groups, there were 79 DE lncRNAs.

LncRNAs have roles in a wide range of physiological and pathological processes, such as glucose and lipid metabolism [39], cancer [40], and skeletal muscle [41] function. Regardless of the BPs of lncRNAs, it is unknown whether lncRNAs function in the control of OS in the piglet jejunum. A total of 79 DE lncRNAs were discovered in our study. Although GO enrichment and KEGG pathway analyses did not reveal direct enrichment of antioxidant pathways, there were many pathways associated with OS, such as chemical carcinogenesis-ROS, colorectal cancer, Foxo pathway, AMPK pathway, and other pathways. Our research lays the groundwork for further study into the involvement of lncRNAs in DQ-induced OS.

Related research has reported that Foxo6 inhibits melanin formation in part by increasing intracellular antioxidant capacity [42] and suppressing ROS production [43]. In this study, the cis-target gene Foxo6 of the DE novel lncRNA MSTRG.13992.1 was enriched in the Foxo signal pathway. The lncRNA MSTRG.13992.1 was highly expressed in the OS group compared with the NC group. In conclusion, this finding predicts that the lncRNA MSTRG.13992.1 may prevent damage to the jejunum caused by OS from DQ.

OSU53, a novel AMPK activator, protects spinal cord nerves from OS caused by hydrogen peroxide (H_2_O_2_) through AMPK signaling pathways [44]. Through activation of AMPK, licochalcone D reduces OS-induced senescence [45]. SIRT1 is a deacetylase that affects gene expression by histone deacetylation. A research on SIRT1 revealed evidence of its function in reducing OS and inflammation [46]. The finding of this research revealed that SIRT1, the trans-target gene of lncRNA MSTRG.3385.1 and ENSSSCG00000049859, was enriched in AMPK signal pathway. Comparison of the expression levels of MSTRG.3385.1 and ENSSSCG00000049859 between the OS and NC groups revealed that the OS group had higher expression of the lncRNA MSTRG.3385.1 and ENSSSCG 00000049859. As the jejunum in the piglets was injected with DQ in the OS group, excessive free radicals were generated. Upregulation of the lncRNAs MSTRG.3385.1 and ENSSSCG 00000049859, the trans-target genes of SIRT1, may have had a role in counteracting the excessive production of free radicals in the OS group. This finding indicates that the upregulation of MSTRG.3385.1 and ENSSSCG00000049859 may fight against OS in the jejunum.

Related studies have reported an association between OS and colorectal cancer. Excessive formation of ROS/RNS leads to OS, which is directly related to the progression of colorectal cancer [47,48]. The trans-target gene SOS1 of the lncRNA MSTRG.5871.1 and ENSSSCG00000042361 was enriched in the colorectal cancer pathway. Compared with the NC group, the OS group exhibited lower expression of the lncRNA MSTRG.5871.1 and ENSSSCG00000042361. For colorectal cancer, this predicts that the downregulation of MSTRG.5871.1 and ENSSSCG00000042361 may provide a viable treatment option. This study’s finding supports previous research in which SOS1 degraders were deemed to be feasible therapeutic agents for KRAS-mutant colorectal cancer [49].

## CONCLUSION

In conclusion, this research discovered that DQ-induced OS caused a difference in the expression of lncRNAs in the jejunum in piglets. In brief, 79 DE lncRNAs were identified. The target genes of the DE lncRNAs were enriched in pathways related to OS. Therefore, there DE lncRNAs were found to have crucial functions in OS. Additionally, our findings establish a solid basis for future research into oxidative-induced pathological processes.

## Figures and Tables

**Table 1:** Primers for quantitative real-time polymerase chain reaction analysis

Gene	Sequence (5′-3′)	Chromosome number	Genomic position
*GAPDH-F*	AAGTTCCACGGCACAGTCAAG		
*GAPDH-R*	CACCAGCATCACCCCATTT		
*MSTRG.13992.1-F*	GAATGTGCTGTCCTCTCCCTTA	6	169769542–169774327
*MSTRG.13992.1-R*	CCTCTATCCTGTGGCTTCATCTAC	6	169769542–169774327
*ENSSSCG00000051217-F*	TCTCCTGGCAAGGTGAGAAGC	14	85421568–85426434
*ENSSSCG00000051217-R*	CATTGCCCGATGCCAGAGAAC	14	85421568–85426434
*ENSSSCG00000042863-F*	CCTGGAACTCTGGGAAACAGGA	5	56447575–56451669
*ENSSSCG00000042863-R*	CCCCTGAAGCCATTCAGCTCT	5	56447575–56451669
*ENSSSCG00000046347-F*	ACCCATGTGTTGCCAAAACTACC	14	67559596–67576058
*ENSSSCG00000046347-R*	TCTCTCCCAAGCATTAGTCTGGA	14	67559596–67576058
*ENSSSCG00000048058-F*	TTTCAGCAGGCACCACACTCT	9	123345691–123457331
*ENSSSCG00000048058-R*	CCTGTGCCACCAAGACTGAGA	9	123345691–123457331
*ENSSSCG00000042361-F*	AGACAATGTTCCTGCCGAAGAA	15	3113891–3179365
*ENSSSCG00000042361-R*	GCCTCAGTCCATCCTCCTCATA	15	3113891–3179365

*GAPDH*, glyceraldehyde-3-phosphate dehydrogenase; F, the forward chain of primers; R, the reverse chain of primers.

**Table 2:** Statistics of sequencing read quality control

Sample	L01	L02	L03	L04	L05	L06
Total reads count (#)	168,319,300	204,605,264	184,167,668	110,986,482	114,567,750	106,287,400
Total bases count (bp)	25,099,860,074	30,490,139,350	27,525,906,846	16,589,901,748	17,103,222,848	15,880,642,184
Average read length (bp)	149	149	149	149	149	149
Q20 bases count (bp)	24,591,604,302	29,804,193,516	26,896,425,748	16,305,157,015	16,796,443,609	15,526,006,867
Q20 bases ratio (%)	97.76	97.75	97.71	98.28	98.20	97.76
Q30 bases count (bp)	23,496,831,051	28,543,214,436	25,737,842,587	15,749,508,935	16,207,042,479	14,875,967,073
Q30 bases ratio (%)	93.61	93.61	93.50	94.93	94.76	93.67
N bases count (bp)	15,482	18,840	16,758	10,250	10,214	9,554
N bases ratio (%)	0.009137	0.009142	0.009041	0.009175	0.008854	0.008916
GC bases ratio (%)	50.71	50.28	50.91	51.12	50.71	51.29

Q20 bases ratio, the percentage of bases in the clean data with quality values greater than or equal to 20; Q30 bases ratio, the percentage of bases in the clean data with quality values greater than or equal to 30; N%, the percentage of undetermined bases in the clean data out of the total bases; GC bases ratio, the percentage of G and C bases out of the total bases.

**Table 3:** Statistics of sequencing read alignment

Sample	L01	L02	L03	L04	L05	L06
Total reads	168,319,300	204,605,264	184,167,668	110,986,482	114,567,750	106,287,400
Uniquely mapped reads	141,478,910	173,369,972	156,844,246	96,754,100	99,599,594	91,982,716
Percentage of uniquely mapped reads (%)	84.05	84.73	85.16	87.18	86.94	86.54
Multiple mapped reads	12,087,902	11,684,728	11,687,670	5,506,124	5,863,598	7,304,724
Percentage of multiple mapped (%)	7.18	5.71	6.35	4.96	5.12	6.87
Unmapped reads	13,110,782	16,796,230	13,586,796	7,551,296	7,800,662	6,355,936
Percentage of unmapped reads (%)	7.79	8.21	7.38	6.80	6.81	5.98
Overall alignment rate (%)	95.49	95.33	95.98	96.11	96.14	96.67

## SUPPLEMENTARY MATERIAL

**Supplementary Table S1**. The list of differentially expressed lncRNAs

**Supplementary Table S2**. The expression of differentially expressed lncRNAs

**Supplementary Table S3**. Top 50 of lncRNA-mRNA gene pairs

**Supplementary Table S4**. GO enrichment analyses of target genes of DE-lncRNAs

**Supplementary Table S5**. KEGG pathways of target genes of DE lncRNAs
