# Supplementary material for: Identification and functional prediction of long non-coding RNAs related to oxidative stress in the jejunum of piglets
Source: Anim Biosci. 2023 Aug 25;37(2):193–202. doi: 10.5713/ab.23.0202 (PMC10766486; doi:10.5713/ab.23.0202)
Supplement: Supplementary file 1 [file ab-23-0202-Supplementary-Table-1.pdf]

Supplementary Material

Supplementary Table 1. The list of differentially expressed lncRNAs

Gene	baseMean	log2FoldChange	lfcSE	stat	pvalue	padj	regulated
ENSSSCG00000044318	89.80085	-3.97111	0.599975	-6.61879	3.62E-11	1.23E-08	DOWN
ENSSSCG00000044182	177.4448	1.863543	0.448331	4.156627	3.23E-05	0.003831	UP
ENSSSCG00000049859	352.3537	2.823185	0.706508	3.995967	6.44E-05	0.006623	UP
ENSSSCG00000049118	42.96954	2.60432	0.595015	4.3769	1.20E-05	0.001954	UP
ENSSSCG00000049680	51.96787	-3.89154	0.903729	-4.30609	1.66E-05	0.002329	DOWN
ENSSSCG00000047532	124.291	-1.8203	0.477512	-3.81205	0.000138	0.011806	DOWN
ENSSSCG00000043234	2277.713	2.995126	0.38248	7.830801	4.85E-15	3.74E-12	UP
ENSSSCG00000043059	59.64674	2.242687	0.662526	3.385058	0.000712	0.045722	UP
ENSSSCG00000040019	348.2246	-1.6551	0.389679	-4.24733	2.16E-05	0.002669	DOWN
ENSSSCG00000045249	62.98017	-3.34043	0.643004	-5.19503	2.05E-07	4.21E-05	DOWN
ENSSSCG00000049915	132.403	2.432211	0.717364	3.390485	0.000698	0.045722	UP
ENSSSCG00000044928	2172.596	2.211467	0.487094	4.540123	5.62E-06	0.00102	UP
ENSSSCG00000042063	60.94509	5.48111	0.864675	6.338926	2.31E-10	6.49E-08	UP
ENSSSCG00000050054	70.50839	-1.91277	0.522947	-3.65767	0.000255	0.019145	DOWN
ENSSSCG00000046347	195.8958	-3.36772	0.43584	-7.72697	1.10E-14	6.79E-12	DOWN
ENSSSCG00000051217	42.26071	6.301833	1.128176	5.585858	2.33E-08	5.52E-06	UP
ENSSSCG00000040730	44.64147	-2.16438	0.625969	-3.45765	0.000545	0.037345	DOWN

ENSSSCG00000045927	47.79958	-3.22723	0.938097	-3.44018	0.000581	0.038974	DOWN
ENSSSCG00000042361	370.0353	-2.09968	0.578061	-3.63229	0.000281	0.020628	DOWN
ENSSSCG00000048058	3810.368	-4.58455	0.502365	-9.12594	7.11E-20	2.19E-16	DOWN
ENSSSCG00000042861	128.4421	-1.94156	0.509261	-3.81251	0.000138	0.011806	DOWN
ENSSSCG00000035711	1053.664	-5.02816	0.717522	-7.00767	2.42E-12	9.34E-10	DOWN
ENSSSCG00000050529	22.67409	-4.13948	1.046665	-3.95493	7.66E-05	0.007616	DOWN
ENSSSCG00000042534	339.1032	1.861457	0.465593	3.99803	6.39E-05	0.006623	UP
ENSSSCG00000045841	131.1105	2.773698	0.768136	3.610946	0.000305	0.021881	UP
ENSSSCG00000043070	75.47355	-1.97269	0.523973	-3.76487	0.000167	0.013524	DOWN
ENSSSCG00000042722	50.68355	2.532483	0.631079	4.01294	6.00E-05	0.006605	UP
ENSSSCG00000041066	581.3033	1.932095	0.493395	3.915916	9.01E-05	0.00868	UP
ENSSSCG00000043684	123.0046	2.129031	0.599337	3.552311	0.000382	0.026765	UP
ENSSSCG00000047615	3964.744	-2.06054	0.55388	-3.7202	0.000199	0.015348	DOWN
ENSSSCG00000030936	43.69906	2.636794	0.605563	4.354288	1.34E-05	0.002059	UP
ENSSSCG00000046312	1298.295	-4.77921	0.547216	-8.73368	2.47E-18	3.80E-15	DOWN
ENSSSCG00000035331	170.6381	2.101462	0.493192	4.260939	2.04E-05	0.002621	UP
ENSSSCG00000038741	545.9608	4.069481	0.576519	7.058716	1.68E-12	7.40E-10	UP
ENSSSCG00000043954	68.54631	3.729406	0.564641	6.604914	3.98E-11	1.23E-08	UP
ENSSSCG00000047869	103.8157	-2.02429	0.521073	-3.88485	0.000102	0.009288	DOWN
ENSSSCG00000047806	28.01162	-3.17426	0.783664	-4.05054	5.11E-05	0.005837	DOWN
ENSSSCG00000042863	283.0652	1.551876	0.461511	3.362594	0.000772	0.048597	UP
ENSSSCG00000045255	166.4251	-2.45969	0.657746	-3.73958	0.000184	0.014576	DOWN
ENSSSCG00000045929	33.23666	-8.24692	1.397492	-5.90123	3.61E-09	9.27E-07	DOWN

ENSSSCG00000047974	217.265	4.904851	0.580906	8.44345	3.08E-17	3.17E-14	UP
ENSSSCG00000043665	225.5911	3.44518	0.785081	4.388313	1.14E-05	0.001954	UP
ENSSSCG00000047628	270.9009	-1.8746	0.480917	-3.89798	9.70E-05	0.009065	DOWN
ENSSSCG00000047695	35.5049	3.98816	0.921158	4.329506	1.49E-05	0.002195	UP
ENSSSCG00000051428	12806.39	-2.64842	0.557324	-4.75203	2.01E-06	0.000388	DOWN
ENSSSCG00000045345	26.07284	5.178347	0.967806	5.350607	8.77E-08	1.93E-05	UP
ENSSSCG00000043117	134.6642	2.035858	0.537028	3.790972	0.00015	0.012508	UP
ENSSSCG00000048590	305.1421	-4.84768	0.631174	-7.68042	1.59E-14	8.15E-12	DOWN
ENSSSCG00000045537	24.07056	-3.63077	0.852191	-4.26051	2.04E-05	0.002621	DOWN
MSTRG.816.1	163.8695	-4.87076	0.65844	-7.39743	1.39E-13	2.65E-11	DOWN
MSTRG.1518.1	45201.47	-2.12692	0.640292	-3.3218	0.000894	0.025275	DOWN
MSTRG.2166.7	12529.81	-1.1637	0.336115	-3.46222	0.000536	0.016992	DOWN
MSTRG.4272.4	796.7499	1.112391	0.286867	3.877729	0.000105	0.005394	UP
MSTRG.3385.1	37.91212	4.297277	1.316931	3.2631	0.001102	0.029193	UP
MSTRG.5135.2	1828.677	-3.52671	0.383322	-9.20038	3.57E-20	2.72E-17	DOWN
MSTRG.5871.1	292.626	-2.55729	0.735363	-3.47759	0.000506	0.016784	DOWN
MSTRG.5937.1	359.3847	4.143397	0.793209	5.223586	1.75E-07	1.67E-05	UP
MSTRG.6368.1	633.4564	-2.64283	0.549001	-4.81389	1.48E-06	0.000125	DOWN
MSTRG.7459.1	331.932	3.546553	0.938071	3.780687	0.000156	0.006281	UP
MSTRG.8911.1	277.2792	-1.35883	0.424478	-3.20119	0.001369	0.033685	DOWN
MSTRG.10024.1	480.2609	1.189745	0.308175	3.860609	0.000113	0.005394	UP
MSTRG.11736.1	168.6875	-1.30431	0.348018	-3.74783	0.000178	0.006492	DOWN
MSTRG.13992.1	733.3372	1.49482	0.475598	3.143034	0.001672	0.03866	UP

MSTRG.13579.2	204.4291	2.43417	0.54812	4.440944	8.96E-06	0.000683	UP
MSTRG.14643.4	1077.968	-1.21586	0.290543	-4.18478	2.85E-05	0.001675	DOWN
MSTRG.15056.1	1717.133	1.341382	0.423186	3.169722	0.001526	0.036382	UP
MSTRG.15498.1	16444.83	-1.34855	0.350875	-3.8434	0.000121	0.005427	DOWN
MSTRG.15466.1	1578.791	1.79529	0.479076	3.747398	0.000179	0.006492	UP
MSTRG.15894.7	50.13572	-2.59591	0.607465	-4.27335	1.93E-05	0.001224	DOWN
MSTRG.16728.1	7099.305	-4.43039	0.496168	-8.92922	4.29E-19	1.64E-16	DOWN
MSTRG.16869.1	335.3336	-2.28515	0.614184	-3.72063	0.000199	0.006892	DOWN
MSTRG.16888.1	6306.75	-2.00455	0.259794	-7.71592	1.20E-14	3.05E-12	DOWN
MSTRG.16888.4	4938.158	-2.69014	0.381203	-7.05697	1.70E-12	2.60E-10	DOWN
MSTRG.16929.1	451.3243	1.845198	0.476618	3.871439	0.000108	0.005394	UP
MSTRG.17089.27	234685.3	-1.59232	0.468641	-3.39774	0.000679	0.019939	DOWN
MSTRG.17090.1	268.9303	-2.7833	0.853469	-3.26117	0.00111	0.029193	DOWN
MSTRG.17149.1	5128.255	-2.14062	0.378463	-5.65609	1.55E-08	1.69E-06	DOWN
MSTRG.17150.27	3675.888	-1.59404	0.365519	-4.36101	1.29E-05	0.000898	DOWN
MSTRG.17339.2	364035.4	-4.15193	0.696009	-5.96534	2.44E-09	3.10E-07	DOWN
