# Supplementary material for: Identification and functional prediction of long non-coding RNAs related to oxidative stress in the jejunum of piglets
Source: Anim Biosci. 2023 Aug 25;37(2):193–202. doi: 10.5713/ab.23.0202 (PMC10766486; doi:10.5713/ab.23.0202)
Supplement: Supplementary file 2 [file ab-23-0202-Supplementary-Table-2.pdf]

Supplementary Table 2. The expression of differentially expressed lncRNAs

Gene	L01	L02	L03	L04	L05	L06
MSTRG.816.1	6.282024	13.64141	15.37762	0.886669	0.426468	0.175989
MSTRG.1518.1	179.6728	276.7197	194.7326	125.4475	21.90765	27.985
MSTRG.2166.7	69.94915	94.00913	38.60456	37.4636	42.73819	27.73133
MSTRG.4272.4	3.259163	4.037964	2.238174	8.434182	9.492395	6.651835
MSTRG.3385.1	0	0.026802	0.013525	0.275423	0.824272	0.025726
MSTRG.5135.2	1.691993	1.411006	1.232588	0.137032	0.11991	0.168784
MSTRG.5871.1	1.106886	1.348558	0.127715	0.147488	0.285357	0.111141
MSTRG.5937.1	0.222366	0.057268	0.062244	0.581394	5.800529	1.715982
MSTRG.6368.1	9.172956	31.42804	7.666214	3.252385	3.108202	2.531547
MSTRG.7459.1	1.581863	0.073736	2.120915	16.49163	10.24266	23.11962
MSTRG.8911.1	100.7304	115.4384	44.77333	36.52432	55.47597	31.28719
MSTRG.10024.1	14.28212	16.05858	15.04957	43.297	39.22888	38.53419
MSTRG.11736.1	39.32737	37.95739	28.07858	18.90326	18.11443	13.8004
MSTRG.13992.1	16.69194	7.101781	10.4153	56.34451	32.23378	27.0888
MSTRG.13579.2	0.753183	0.62526	0.466115	2.891965	2.809486	5.217212
MSTRG.14643.4	2.284616	3.829867	2.540998	1.258357	1.885998	1.308871
MSTRG.15056.1	0	0.002712	0.013683	0.027319	0.014016	0.065068
MSTRG.15498.1	281.7559	342.4215	232.766	142.4887	104.6591	135.1858
MSTRG.15466.1	1.211932	2.520949	4.555482	21.67288	14.70554	4.64738
MSTRG.15894.7	0.542806	0.472614	0.814633	0.139099	0.137238	0.093445
MSTRG.16728.1	16.11475	14.63928	4.194016	0.63206	1.058259	0.358581

MSTRG.16869.1	117.0022	158.8418	41.57272	10.54831	46.28461	23.14051
MSTRG.16888.1	19.71944	22.3715	27.29521	5.36918	9.92061	4.59207
MSTRG.16888.4	122.5977	271.8206	147.4999	23.4181	43.284	32.33357
MSTRG.16929.1	0.767546	2.378578	1.255106	6.784202	5.501575	5.803203
MSTRG.17089.27	25.17189	25.92029	45.06409	10.71778	27.73606	7.404088
MSTRG.17090.1	0.580338	0.871337	3.036383	0.073154	0.719348	0.135519
MSTRG.17149.1	31.43645	52.93025	24.02068	6.49774	16.09082	7.725579
MSTRG.17150.27	4.108905	4.524459	2.350158	1.44141	2.592081	0.660675
MSTRG.17339.2	10808.08	12323.7	8169.522	1404.176	151.5433	436.7017
ENSSSCG00000044318	1.736774	1.879422	0.822441	0.076207	0.090842	0.152773
ENSSSCG00000044182	4.604266	3.203478	4.58034	19.84072	17.27959	11.1798
ENSSSCG00000049859	0.290421	0.243574	0.351581	0.363687	5.924486	0.869712
ENSSSCG00000049118	0.102605	0.076312	0.166122	0.678539	0.684148	0.886063
ENSSSCG00000049680	6.877832	28.23288	6.240778	0.699754	0	2.454896
ENSSSCG00000047532	11.48642	17.08597	8.589587	3.069937	4.515705	4.142319
ENSSSCG00000043234	3.302633	1.918271	3.506537	28.01164	23.6557	23.0921
ENSSSCG00000043059	1.150845	0.454717	0.831816	7.039894	2.381463	2.614379
ENSSSCG00000040019	7.384339	6.053674	5.547587	2.203021	1.914853	2.490357
ENSSSCG00000045249	8.889509	19.83463	14.02346	1.356635	0.628896	2.644101
ENSSSCG00000049915	1.566472	1.165058	4.1213	4.07897	31.80746	4.769982
ENSSSCG00000044928	1.914362	0.677865	2.26504	10.76347	5.679088	7.22832
ENSSSCG00000042063	0.022287	0	0.185572	4.392107	1.414303	3.37815
ENSSSCG00000050054	21.62613	12.06327	16.41665	3.66709	5.92859	5.145991

ENSSSCG00000046347	19.87103	13.53446	14.97815	1.604583	1.673633	1.876413
ENSSSCG00000051217	0	0	0.523666	21.52334	16.98358	2.188659
ENSSSCG00000040730	0.835088	0.621094	1.790461	0.262979	0.256009	0.246023
ENSSSCG00000045927	3.547471	2.731322	0.485367	0	0.206784	0.656876
ENSSSCG00000042361	7.184242	10.96815	1.819794	1.808594	1.349518	2.049908
ENSSSCG00000048058	70.67779	53.36929	18.56592	2.178516	2.765865	1.746091
ENSSSCG00000042861	13.18265	6.976316	7.544419	1.564745	3.963625	2.614037
ENSSSCG00000035711	24.6344	1.357705	5.287934	0.25823	0.437681	0.407668
ENSSSCG00000050529	3.730818	16.6487	5.52261	0.986896	0	0.577042
ENSSSCG00000042534	5.349097	5.15294	14.65791	27.69303	42.58919	26.79006
ENSSSCG00000045841	1.225349	0.67767	1.569673	3.03358	1.387014	21.86812
ENSSSCG00000043070	13.15532	11.23119	11.82804	3.308373	2.044884	4.728581
ENSSSCG00000042722	0.157096	0.423544	0.381518	1.740153	1.191965	3.128356
ENSSSCG00000041066	1.629834	1.700504	2.85852	3.749422	15.85621	6.570935
ENSSSCG00000043684	3.944424	1.490109	3.996801	12.0902	26.94841	6.100807
ENSSSCG00000047615	301.6211	148.0256	126.791	41.79219	19.41694	93.54774
ENSSSCG00000030936	0.075181	0.121997	0.069555	0.705091	0.565718	0.539122
ENSSSCG00000046312	6.448342	29.65053	37.35072	0.587792	0.877556	1.415172
ENSSSCG00000035331	7.515292	5.876112	6.863763	13.1258	45.19243	39.64026
ENSSSCG00000038741	0.230806	0.141807	0.306375	2.282893	8.776967	1.56246
ENSSSCG00000043954	0.245753	0.264013	0.353675	5.092286	4.181333	2.91413
ENSSSCG00000047869	3.712994	5.050084	2.201527	1.139547	0.962188	0.856669
ENSSSCG00000047806	1.709804	1.759131	1.160027	0.075381	0.15725	0.352605

ENSSSCG00000042863	26.23653	19.7635	33.76463	69.62475	50.34741	133.7053
ENSSSCG00000045255	1.559386	1.409443	0.868761	0.073058	0.117234	0.611187
ENSSSCG00000045929	22.89591	57.5572	17.36969	0	0	0
ENSSSCG00000047974	0.045217	0.055031	0.091272	0.907948	4.128324	1.328791
ENSSSCG00000043665	0.00462	0.106523	0.188074	0.467471	2.453872	0.603832
ENSSSCG00000047628	5.903857	3.727527	6.78685	1.98708	2.007821	0.780621
ENSSSCG00000047695	2.00259	0	0.50529	13.72498	7.032229	22.24488
ENSSSCG00000051428	836.0435	1463.273	1504.083	198.8032	56.87954	396.8341
ENSSSCG00000045345	0.233092	0	0.143766	2.774644	6.002464	5.527993
ENSSSCG00000043117	5.023814	6.808632	7.539876	16.24243	17.55745	53.874
ENSSSCG00000048590	18.90353	76.48751	40.78574	0.559753	1.167682	3.600191
ENSSSCG00000045537	4.089161	9.240872	9.894414	0.62405	0.433937	0.973026
