# Supplementary material for: Identification and functional prediction of long non-coding RNAs related to oxidative stress in the jejunum of piglets
Source: Anim Biosci. 2023 Aug 25;37(2):193–202. doi: 10.5713/ab.23.0202 (PMC10766486; doi:10.5713/ab.23.0202)
Supplement: Supplementary file 3 [file ab-23-0202-Supplementary-Table-3.pdf]

Supplementary Table 3. Top 50 of lncRNA-mRNA gene pairs

gene1(lncRNA)	gene2(mRNA)	gene2Type	cor	pvalue
ENSSSCG00000042722	PC	protein_coding	1	5.61E-08
ENSSSCG00000045255	LPCAT1	protein_coding	-1	8.01E-08
MSTRG.16929.1	ZNF444	protein_coding	1	2.59E-07
ENSSSCG00000048058	PCNX3	protein_coding	-0.999	9.36E-07
ENSSSCG00000045841	SPTBN1	protein_coding	0.999	1.58E-06
ENSSSCG00000038741	PTGR1	protein_coding	-0.999	1.61E-06
ENSSSCG00000047974	ENSSSCG00000036046	protein_coding	0.999	1.80E-06
ENSSSCG00000046347	GKN1	protein_coding	0.999	1.81E-06
ENSSSCG00000049915	SLC7A6OS	protein_coding	0.999	2.19E-06
MSTRG.3385.1	HAND1	protein_coding	0.999	2.27E-06
ENSSSCG00000043665	BRPF1	protein_coding	0.999	2.31E-06
MSTRG.3385.1	LOC100519606	protein_coding	0.999	2.38E-06
ENSSSCG00000049859	HSPB3	protein_coding	0.999	2.49E-06
MSTRG.816.1	TXNDC11	protein_coding	0.999	2.65E-06
ENSSSCG00000045345	RCN2	protein_coding	-0.999	3.05E-06
ENSSSCG00000049859	DDX23	protein_coding	0.998	3.42E-06
ENSSSCG00000043234	NHSL2	protein_coding	0.998	3.50E-06
MSTRG.3385.1	FOXI3	protein_coding	0.998	3.59E-06
ENSSSCG00000045927	CCDC106	protein_coding	-0.998	3.61E-06
MSTRG.3385.1	ENSSSCG00000043564	protein_coding	0.998	3.68E-06
ENSSSCG00000049859	ENSSSCG00000047152	protein_coding	0.998	3.76E-06
ENSSSCG00000049118	LRRC29	protein_coding	0.998	3.81E-06
ENSSSCG00000041066	ZNF287	protein_coding	0.998	3.90E-06

ENSSSCG00000049915	C6H1orf174	protein_coding	0.998	4.16E-06
ENSSSCG00000049859	MYBL2	protein_coding	0.998	4.79E-06
ENSSSCG00000043234	LOC100522551	protein_coding	0.998	4.80E-06
ENSSSCG00000049915	DDX55	protein_coding	0.998	4.84E-06
MSTRG.16869.1	NARS1	protein_coding	0.998	5.06E-06
ENSSSCG00000045255	ENSSSCG00000047285	protein_coding	-0.998	5.40E-06
MSTRG.5135.2	TRIOBP	protein_coding	-0.998	5.81E-06
ENSSSCG00000049859	BICRAL	protein_coding	0.998	5.88E-06
ENSSSCG00000051428	ZNF143	protein_coding	-0.998	5.93E-06
ENSSSCG00000049859	TRNAU1AP	protein_coding	0.998	6.02E-06
ENSSSCG00000049859	ELFN2	protein_coding	0.998	6.04E-06
ENSSSCG00000047806	MAGT1	protein_coding	0.998	6.19E-06
MSTRG.5937.1	LOC100512873	protein_coding	0.998	6.36E-06
ENSSSCG00000042534	CBARP	protein_coding	0.998	6.89E-06
ENSSSCG00000040019	NLE1	protein_coding	-0.998	7.70E-06
MSTRG.13579.2	GABRD	protein_coding	0.998	7.71E-06
MSTRG.17089.27	DOP1B	protein_coding	-0.998	7.85E-06
ENSSSCG00000043070	GSTO1	protein_coding	0.998	8.20E-06
ENSSSCG00000038741	GK5	protein_coding	-0.998	8.25E-06
ENSSSCG00000047615	GCSH	protein_coding	0.998	8.31E-06
ENSSSCG00000041066	LOC100627241	protein_coding	0.998	8.34E-06
ENSSSCG00000047806	NCOR1	protein_coding	-0.998	8.47E-06
ENSSSCG00000049859	ISG20L2	protein_coding	0.998	8.83E-06
MSTRG.17090.1	C6H16orf78	protein_coding	0.998	9.02E-06
MSTRG.15056.1	ETV3	protein_coding	0.997	9.42E-06

ENSSSCG00000049915	MOB3A	protein_coding	0.997	9.76E-06
ENSSSCG00000042063	MAP4K5	protein_coding	-0.997	1.01E-05
