# Supplementary material for: Identification and functional prediction of long non-coding RNAs related to oxidative stress in the jejunum of piglets
Source: Anim Biosci. 2023 Aug 25;37(2):193–202. doi: 10.5713/ab.23.0202 (PMC10766486; doi:10.5713/ab.23.0202)
Supplement: Supplementary file 4 [file ab-23-0202-Supplementary-Table-4.pdf]

Supplementary Table 4. GO enrichment analyses of target genes of DE-lncRNAs

Category	Term	Count	PValue	FDR
GOTERM_BP_DIRECT	GO:0042113~B cell activation	14	1.41E-06	0.008386
GOTERM_BP_DIRECT	GO:0090110~cargo loading into COPII-coated vesicle	11	1.66E-04	0.368633
GOTERM_BP_DIRECT	GO:0009887~animal organ morphogenesis	31	1.86E-04	0.368633
GOTERM_BP_DIRECT	GO:0006888~ER to Golgi vesicle-mediated transport	37	3.10E-04	0.4612
GOTERM_BP_DIRECT	GO:0007049~cell cycle	47	9.57E-04	1
GOTERM_BP_DIRECT	GO:0000278~mitotic cell cycle	31	0.001614	1
GOTERM_BP_DIRECT	GO:0006281~DNA repair	50	0.001754	1
GOTERM_BP_DIRECT	GO:0071277~cellular response to calcium ion	25	0.002483	1
GOTERM_BP_DIRECT	GO:0043304~regulation of mast cell degranulation	7	0.002717	1
GOTERM_BP_DIRECT	GO:0042552~myelination	17	0.003707	1
GOTERM_BP_DIRECT	GO:0009888~tissue development	14	0.003722	1
GOTERM_BP_DIRECT	GO:0044772~mitotic cell cycle phase transition	12	0.005231	1
GOTERM_BP_DIRECT	GO:0040008~regulation of growth	12	0.005231	1
GOTERM_BP_DIRECT	GO:0006310~DNA recombination	17	0.007023	1
GOTERM_BP_DIRECT	GO:0030890~positive regulation of B cell proliferation	16	0.007367	1
GOTERM_BP_DIRECT	GO:0007030~Golgi organization	31	0.008527	1
GOTERM_BP_DIRECT	GO:0022407~regulation of cell-cell adhesion	6	0.008964	1
GOTERM_BP_DIRECT	GO:0000727~double-strand break repair via break-induced replication	8	0.009004	1

GOTERM_BP_DIRECT	GO:0043923~positive regulation by host of viral transcription	9	0.010533	1
GOTERM_BP_DIRECT	GO:0006099~tricarboxylic acid cycle	13	0.01143	1
GOTERM_BP_DIRECT	GO:0035556~intracellular signal transduction	103	0.011936	1
GOTERM_BP_DIRECT	GO:0043087~regulation of GTPase activity	25	0.012328	1
GOTERM_BP_DIRECT	GO:0042267~natural killer cell mediated cytotoxicity	7	0.013207	1
GOTERM_BP_DIRECT	GO:0071805~potassium ion transmembrane transport	26	0.013628	1
GOTERM_BP_DIRECT	GO:0021987~cerebral cortex development	15	0.014177	1
GOTERM_BP_DIRECT	GO:0006357~regulation of transcription from RNA polymerase II promoter	310	0.01526	1
GOTERM_BP_DIRECT	GO:0060766~negative regulation of androgen receptor signaling pathway	8	0.01566	1
GOTERM_BP_DIRECT	GO:0000070~mitotic sister chromatid segregation	11	0.017077	1
GOTERM_BP_DIRECT	GO:0007099~centriole replication	11	0.017077	1
GOTERM_BP_DIRECT	GO:0019228~neuronal action potential	11	0.017077	1
GOTERM_BP_DIRECT	GO:0090398~cellular senescence	10	0.017235	1
GOTERM_BP_DIRECT	GO:0006260~DNA replication	25	0.018059	1
GOTERM_BP_DIRECT	GO:0042493~response to drug	22	0.019905	1
GOTERM_BP_DIRECT	GO:0016567~protein ubiquitination	71	0.021556	1
GOTERM_BP_DIRECT	GO:0045892~negative regulation of transcription, DNA-templated	72	0.023791	1
GOTERM_BP_DIRECT	GO:0019369~arachidonic acid metabolic process	8	0.025172	1

GOTERM_BP_DIRECT	GO:0045736~negative regulation of cyclin-dependent protein serine/threonine kinase activity	9	0.025517	1
GOTERM_BP_DIRECT	GO:0048511~rhythmic process	14	0.026344	1
GOTERM_BP_DIRECT	GO:0033572~transferrin transport	5	0.028313	1
GOTERM_BP_DIRECT	GO:1904668~positive regulation of ubiquitin protein ligase activity	5	0.028313	1
GOTERM_BP_DIRECT	GO:0006750~glutathione biosynthetic process	5	0.028313	1
GOTERM_BP_DIRECT	GO:0039529~RIG-I signaling pathway	5	0.028313	1
GOTERM_BP_DIRECT	GO:0006897~endocytosis	36	0.028608	1
GOTERM_BP_DIRECT	GO:0032088~negative regulation of NF-kappaB transcription factor activity	23	0.031318	1
GOTERM_BP_DIRECT	GO:0000077~DNA damage checkpoint	11	0.032955	1
GOTERM_BP_DIRECT	GO:0031623~receptor internalization	14	0.033877	1
GOTERM_BP_DIRECT	GO:0006974~cellular response to DNA damage stimulus	38	0.034487	1
GOTERM_BP_DIRECT	GO:0098656~anion transmembrane transport	6	0.035396	1
GOTERM_BP_DIRECT	GO:0010569~regulation of double-strand break repair via homologous recombination	9	0.036841	1
GOTERM_BP_DIRECT	GO:0042572~retinol metabolic process	9	0.036841	1
GOTERM_BP_DIRECT	GO:0000079~regulation of cyclin-dependent protein serine/threonine kinase activity	13	0.037008	1
GOTERM_BP_DIRECT	GO:0051973~positive regulation of telomerase activity	13	0.037008	1
GOTERM_BP_DIRECT	GO:0043161~proteasome-mediated ubiquitin-dependent protein catabolic process	41	0.037139	1

GOTERM_BP_DIRECT	GO:0006622~protein targeting to lysosome	8	0.037982	1
GOTERM_BP_DIRECT	GO:0007265~Ras protein signal transduction	15	0.038913	1
GOTERM_BP_DIRECT	GO:0032212~positive regulation of telomere maintenance via telomerase	12	0.04035	1
GOTERM_BP_DIRECT	GO:0045944~positive regulation of transcription from RNA polymerase II promoter	173	0.040423	1
GOTERM_BP_DIRECT	GO:0003093~regulation of glomerular filtration	4	0.040624	1
GOTERM_BP_DIRECT	GO:0006817~phosphate ion transport	4	0.040624	1
GOTERM_BP_DIRECT	GO:0042490~mechanoreceptor differentiation	4	0.040624	1
GOTERM_BP_DIRECT	GO:0001932~regulation of protein phosphorylation	11	0.043871	1
GOTERM_BP_DIRECT	GO:0045732~positive regulation of protein catabolic process	19	0.046875	1
GOTERM_BP_DIRECT	GO:0030282~bone mineralization	13	0.047143	1
GOTERM_BP_DIRECT	GO:0009411~response to UV	13	0.047143	1
GOTERM_BP_DIRECT	GO:0006342~chromatin silencing	13	0.047143	1
GOTERM_BP_DIRECT	GO:0034765~regulation of ion transmembrane transport	32	0.047368	1
GOTERM_BP_DIRECT	GO:0005977~glycogen metabolic process	10	0.0475	1
GOTERM_BP_DIRECT	GO:0031297~replication fork processing	10	0.0475	1
GOTERM_BP_DIRECT	GO:0006270~DNA replication initiation	9	0.0511	1
GOTERM_BP_DIRECT	GO:0035264~multicellular organism growth	24	0.053152	1
GOTERM_BP_DIRECT	GO:0007052~mitotic spindle organization	14	0.053327	1
GOTERM_BP_DIRECT	GO:0097186~amelogenesis	5	0.054111	1
GOTERM_BP_DIRECT	GO:0001955~blood vessel maturation	5	0.054111	1

GOTERM_BP_DIRECT	GO:0043276~anoikis	5	0.054111	1
GOTERM_BP_DIRECT	GO:0071281~cellular response to iron ion	5	0.054111	1
GOTERM_BP_DIRECT	GO:0010032~meiotic chromosome condensation	5	0.054111	1
GOTERM_BP_DIRECT	GO:0051451~myoblast migration	5	0.054111	1
GOTERM_BP_DIRECT	GO:1902667~regulation of axon guidance	5	0.054111	1
GOTERM_BP_DIRECT	GO:0071786~endoplasmic reticulum tubular network organization	5	0.054111	1
GOTERM_BP_DIRECT	GO:0044829~positive regulation by host of viral genome replication	5	0.054111	1
GOTERM_BP_DIRECT	GO:0010918~positive regulation of mitochondrial membrane potential	5	0.054111	1
GOTERM_BP_DIRECT	GO:0060218~hematopoietic stem cell differentiation	7	0.056917	1
GOTERM_BP_DIRECT	GO:0015701~bicarbonate transport	7	0.056917	1
GOTERM_BP_DIRECT	GO:0007064~mitotic sister chromatid cohesion	7	0.056917	1
GOTERM_BP_DIRECT	GO:0045860~positive regulation of protein kinase activity	11	0.056996	1
GOTERM_BP_DIRECT	GO:0016446~somatic hypermutation of immunoglobulin genes	6	0.057586	1
GOTERM_BP_DIRECT	GO:0001833~inner cell mass cell proliferation	6	0.057586	1
GOTERM_BP_DIRECT	GO:0042551~neuron maturation	6	0.057586	1
GOTERM_BP_DIRECT	GO:0006978~DNA damage response, signal transduction by p53 class mediator resulting in transcription of p21 class mediator	6	0.057586	1

GOTERM_BP_DIRECT	GO:0006890~retrograde vesicle-mediated transport, Golgi to ER	15	0.058932	1
GOTERM_BP_DIRECT	GO:0030334~regulation of cell migration	27	0.059481	1
GOTERM_BP_DIRECT	GO:0016573~histone acetylation	10	0.06255	1
GOTERM_BP_DIRECT	GO:0051209~release of sequestered calcium ion into cytosol	10	0.06255	1
GOTERM_BP_DIRECT	GO:0032467~positive regulation of cytokinesis	10	0.06255	1
GOTERM_BP_DIRECT	GO:0007094~mitotic spindle assembly checkpoint	10	0.06255	1
GOTERM_BP_DIRECT	GO:0034394~protein localization to cell surface	10	0.06255	1
GOTERM_BP_DIRECT	GO:0048286~lung alveolus development	12	0.065375	1
GOTERM_BP_DIRECT	GO:0045893~positive regulation of transcription, DNA-templated	69	0.06587	1
GOTERM_BP_DIRECT	GO:0044782~cilium organization	9	0.068472	1
GOTERM_BP_DIRECT	GO:0031146~SCF-dependent proteasomal ubiquitin-dependent protein catabolic process	17	0.068583	1
GOTERM_BP_DIRECT	GO:0050853~B cell receptor signaling pathway	15	0.071162	1
GOTERM_BP_DIRECT	GO:0030032~lamellipodium assembly	11	0.072443	1
GOTERM_BP_DIRECT	GO:1903861~positive regulation of dendrite extension	8	0.074633	1
GOTERM_BP_DIRECT	GO:0006261~DNA-dependent DNA replication	8	0.074633	1
GOTERM_BP_DIRECT	GO:0051301~cell division	40	0.075277	1
GOTERM_BP_DIRECT	GO:0032091~negative regulation of protein binding	19	0.076465	1
GOTERM_BP_DIRECT	GO:0051865~protein autoubiquitination	20	0.07985	1
GOTERM_BP_DIRECT	GO:0050776~regulation of immune response	10	0.080307	1

GOTERM_BP_DIRECT	GO:0060088~auditory receptor cell stereocilium organization	7	0.080726	1
GOTERM_BP_DIRECT	GO:2000773~negative regulation of cellular senescence	7	0.080726	1
GOTERM_BP_DIRECT	GO:0043968~histone H2A acetylation	7	0.080726	1
GOTERM_BP_DIRECT	GO:0001829~trophectodermal cell differentiation	7	0.080726	1
GOTERM_BP_DIRECT	GO:0051402~neuron apoptotic process	12	0.080977	1
GOTERM_BP_DIRECT	GO:0007095~mitotic G2 DNA damage checkpoint	12	0.080977	1
GOTERM_BP_DIRECT	GO:0050859~negative regulation of B cell receptor signaling pathway	4	0.084238	1
GOTERM_BP_DIRECT	GO:0036150~phosphatidylserine acyl-chain remodeling	4	0.084238	1
GOTERM_BP_DIRECT	GO:2000766~negative regulation of cytoplasmic translation	4	0.084238	1
GOTERM_BP_DIRECT	GO:0002532~production of molecular mediator involved in inflammatory response	4	0.084238	1
GOTERM_BP_DIRECT	GO:0090344~negative regulation of cell aging	4	0.084238	1
GOTERM_BP_DIRECT	GO:0035519~protein K29-linked ubiquitination	4	0.084238	1
GOTERM_BP_DIRECT	GO:0060440~trachea formation	4	0.084238	1
GOTERM_BP_DIRECT	GO:0019464~glycine decarboxylation via glycine cleavage system	4	0.084238	1
GOTERM_BP_DIRECT	GO:0006271~DNA strand elongation involved in DNA replication	4	0.084238	1
GOTERM_BP_DIRECT	GO:0051573~negative regulation of histone H3-K9 methylation	4	0.084238	1
GOTERM_BP_DIRECT	GO:0050913~sensory perception of bitter taste	4	0.084238	1

GOTERM_BP_DIRECT	GO:0090114~COPII-coated vesicle budding	4	0.084238	1
GOTERM_BP_DIRECT	GO:0006874~cellular calcium ion homeostasis	22	0.085688	1
GOTERM_BP_DIRECT	GO:0007097~nuclear migration	6	0.086041	1
GOTERM_BP_DIRECT	GO:0071380~cellular response to prostaglandin E stimulus	6	0.086041	1
GOTERM_BP_DIRECT	GO:0022010~central nervous system myelination	6	0.086041	1
GOTERM_BP_DIRECT	GO:0030851~granulocyte differentiation	6	0.086041	1
GOTERM_BP_DIRECT	GO:0043922~negative regulation by host of viral transcription	6	0.086041	1
GOTERM_BP_DIRECT	GO:0031952~regulation of protein autophosphorylation	5	0.088848	1
GOTERM_BP_DIRECT	GO:0045577~regulation of B cell differentiation	5	0.088848	1
GOTERM_BP_DIRECT	GO:0000002~mitochondrial genome maintenance	5	0.088848	1
GOTERM_BP_DIRECT	GO:0019372~lipoxygenase pathway	5	0.088848	1
GOTERM_BP_DIRECT	GO:0001952~regulation of cell-matrix adhesion	5	0.088848	1
GOTERM_BP_DIRECT	GO:1900264~positive regulation of DNA-directed DNA polymerase activity	5	0.088848	1
GOTERM_BP_DIRECT	GO:0010800~positive regulation of peptidyl-threonine phosphorylation	9	0.089037	1
GOTERM_BP_DIRECT	GO:0008654~phospholipid biosynthetic process	9	0.089037	1
GOTERM_BP_DIRECT	GO:0008284~positive regulation of cell proliferation	79	0.089971	1
GOTERM_BP_DIRECT	GO:0016197~endosomal transport	16	0.089982	1
GOTERM_BP_DIRECT	GO:0046326~positive regulation of glucose import	11	0.090272	1
GOTERM_BP_DIRECT	GO:0006284~base-excision repair	11	0.090272	1
GOTERM_BP_DIRECT	GO:0042127~regulation of cell proliferation	32	0.092754	1

GOTERM_BP_DIRECT	GO:0007059~chromosome segregation	18	0.098383	1
GOTERM_BP_DIRECT	GO:0045773~positive regulation of axon extension	8	0.098683	1
GOTERM_BP_DIRECT	GO:0018279~protein N-linked glycosylation via asparagine	8	0.098683	1
GOTERM_BP_DIRECT	GO:0032147~activation of protein kinase activity	12	0.0987	1
GOTERM_CC_DIRECT	GO:0005634~nucleus	913	1.38E-09	1.42E-06
GOTERM_CC_DIRECT	GO:0005829~cytosol	692	3.86E-09	1.98E-06
GOTERM_CC_DIRECT	GO:0005654~nucleoplasm	534	9.63E-08	3.29E-05
GOTERM_CC_DIRECT	GO:0005737~cytoplasm	820	4.84E-07	1.24E-04
GOTERM_CC_DIRECT	GO:0000776~kinetochore	37	3.58E-05	0.007354
GOTERM_CC_DIRECT	GO:0005789~endoplasmic reticulum membrane	135	8.97E-05	0.015345
GOTERM_CC_DIRECT	GO:0000922~spindle pole	34	1.46E-04	0.021348
GOTERM_CC_DIRECT	GO:0000785~chromatin	73	6.19E-04	0.072114
GOTERM_CC_DIRECT	GO:0031901~early endosome membrane	30	6.33E-04	0.072114
GOTERM_CC_DIRECT	GO:0005813~centrosome	120	0.001076	0.11037
GOTERM_CC_DIRECT	GO:0005794~Golgi apparatus	186	0.00174	0.142863
GOTERM_CC_DIRECT	GO:0031390~Ctf18 RFC-like complex	7	0.001789	0.142863
GOTERM_CC_DIRECT	GO:0005657~replication fork	13	0.00181	0.142863
GOTERM_CC_DIRECT	GO:0005814~centriole	38	0.002351	0.172265
GOTERM_CC_DIRECT	GO:0005811~lipid particle	28	0.002593	0.17735
GOTERM_CC_DIRECT	GO:0005739~mitochondrion	207	0.005118	0.328195
GOTERM_CC_DIRECT	GO:0030127~COPII vesicle coat	8	0.005802	0.350145
GOTERM_CC_DIRECT	GO:0005663~DNA replication factor C complex	5	0.008676	0.494534
GOTERM_CC_DIRECT	GO:0005815~microtubule organizing center	31	0.00964	0.52057

GOTERM_CC_DIRECT	GO:0000307~cyclin-dependent protein kinase holoenzyme complex	13	0.011947	0.596152
GOTERM_CC_DIRECT	GO:0005730~nucleolus	150	0.012523	0.596152
GOTERM_CC_DIRECT	GO:0005801~cis-Golgi network	15	0.012783	0.596152
GOTERM_CC_DIRECT	GO:0016607~nuclear speck	85	0.014391	0.633904
GOTERM_CC_DIRECT	GO:0001650~fibrillar center	39	0.014854	0.633904
GOTERM_CC_DIRECT	GO:0000139~Golgi membrane	67	0.015622	0.633904
GOTERM_CC_DIRECT	GO:0097431~mitotic spindle pole	13	0.01611	0.633904
GOTERM_CC_DIRECT	GO:0005694~chromosome	37	0.016682	0.633904
GOTERM_CC_DIRECT	GO:0000775~chromosome, centromeric region	14	0.018805	0.682017
GOTERM_CC_DIRECT	GO:0036464~cytoplasmic ribonucleoprotein granule	19	0.019277	0.682017
GOTERM_CC_DIRECT	GO:0016020~membrane	203	0.025256	0.863769
GOTERM_CC_DIRECT	GO:0098978~glutamatergic synapse	61	0.026377	0.87299
GOTERM_CC_DIRECT	GO:0005759~mitochondrial matrix	35	0.030945	0.992097
GOTERM_CC_DIRECT	GO:0005960~glycine cleavage complex	4	0.032877	0.992097
GOTERM_CC_DIRECT	GO:0000811~GINS complex	4	0.032877	0.992097
GOTERM_CC_DIRECT	GO:0043197~dendritic spine	23	0.037758	0.992263
GOTERM_CC_DIRECT	GO:0000791~euchromatin	12	0.040297	0.992263
GOTERM_CC_DIRECT	GO:0035097~histone methyltransferase complex	6	0.04288	0.992263
GOTERM_CC_DIRECT	GO:0072687~meiotic spindle	6	0.04288	0.992263
GOTERM_CC_DIRECT	GO:0030877~beta-catenin destruction complex	6	0.04288	0.992263
GOTERM_CC_DIRECT	GO:0016604~nuclear body	58	0.045541	0.992263
GOTERM_CC_DIRECT	GO:0000781~chromosome, telomeric region	24	0.050642	0.992263

GOTERM_CC_DIRECT	GO:0030134~ER to Golgi transport vesicle	12	0.050757	0.992263
GOTERM_CC_DIRECT	GO:0005802~trans-Golgi network	40	0.055791	0.992263
GOTERM_CC_DIRECT	GO:0030424~axon	52	0.06412	0.992263
GOTERM_CC_DIRECT	GO:0042555~MCM complex	6	0.065077	0.992263
GOTERM_CC_DIRECT	GO:0000812~Swr1 complex	6	0.065077	0.992263
GOTERM_CC_DIRECT	GO:0008250~oligosaccharyltransferase complex	6	0.065077	0.992263
GOTERM_CC_DIRECT	GO:0031965~nuclear membrane	41	0.065957	0.992263
GOTERM_CC_DIRECT	GO:0031410~cytoplasmic vesicle	46	0.065976	0.992263
GOTERM_CC_DIRECT	GO:0051233~spindle midzone	10	0.067816	0.992263
GOTERM_CC_DIRECT	GO:0030663~COPI-coated vesicle membrane	4	0.069177	0.992263
GOTERM_CC_DIRECT	GO:0097124~cyclin A2-CDK2 complex	4	0.069177	0.992263
GOTERM_CC_DIRECT	GO:0097539~ciliary transition fiber	5	0.069948	0.992263
GOTERM_CC_DIRECT	GO:1990124~messenger ribonucleoprotein complex	5	0.069948	0.992263
GOTERM_CC_DIRECT	GO:0000796~condensin complex	5	0.069948	0.992263
GOTERM_CC_DIRECT	GO:0032133~chromosome passenger complex	5	0.069948	0.992263
GOTERM_CC_DIRECT	GO:0035267~NuA4 histone acetyltransferase complex	8	0.070259	0.992263
GOTERM_CC_DIRECT	GO:0019005~SCF ubiquitin ligase complex	19	0.074606	0.992263
GOTERM_CC_DIRECT	GO:0030136~clathrin-coated vesicle	13	0.079684	0.992263
GOTERM_CC_DIRECT	GO:0031616~spindle pole centrosome	7	0.080727	0.992263
GOTERM_CC_DIRECT	GO:0070062~extracellular exosome	21	0.085051	0.992263
GOTERM_CC_DIRECT	GO:0005793~endoplasmic reticulum-Golgi intermediate compartment	18	0.085423	0.992263
GOTERM_CC_DIRECT	GO:0005769~early endosome	45	0.092274	0.992263

GOTERM_CC_DIRECT	GO:0031430~M band	6	0.0925	0.992263
GOTERM_CC_DIRECT	GO:0030665~clathrin-coated vesicle membrane	6	0.0925	0.992263
GOTERM_CC_DIRECT	GO:0030054~cell junction	30	0.094883	0.992263
GOTERM_CC_DIRECT	GO:0030496~midbody	35	0.095235	0.992263
GOTERM_CC_DIRECT	GO:0016363~nuclear matrix	19	0.09738	0.992263
GOTERM_CC_DIRECT	GO:0042470~melanosome	16	0.097738	0.992263
GOTERM_CC_DIRECT	GO:0030173~integral component of Golgi membrane	16	0.097738	0.992263
GOTERM_CC_DIRECT	GO:0099056~integral component of presynaptic membrane	9	0.098871	0.992263
GOTERM_MF_DIRECT	GO:0046872~metal ion binding	397	6.81E-05	0.135534
GOTERM_MF_DIRECT	GO:0000978~RNA polymerase II core promoter proximal region sequence-specific DNA binding	255	1.35E-04	0.135534
GOTERM_MF_DIRECT	GO:0003682~chromatin binding	104	2.08E-04	0.139912
GOTERM_MF_DIRECT	GO:0008270~zinc ion binding	186	6.85E-04	0.336868
GOTERM_MF_DIRECT	GO:0000981~RNA polymerase II transcription factor activity, sequence-specific DNA binding	235	9.77E-04	0.336868
GOTERM_MF_DIRECT	GO:0003714~transcription corepressor activity	46	0.001003	0.336868
GOTERM_MF_DIRECT	GO:0003677~DNA binding	258	0.001733	0.417651
GOTERM_MF_DIRECT	GO:0003689~DNA clamp loader activity	7	0.001865	0.417651
GOTERM_MF_DIRECT	GO:0008194~UDP-glycosyltransferase activity	7	0.001865	0.417651
GOTERM_MF_DIRECT	GO:0008327~methyl-CpG binding	12	0.004617	0.780929
GOTERM_MF_DIRECT	GO:0005524~ATP binding	332	0.004651	0.780929
GOTERM_MF_DIRECT	GO:0010997~anaphase-promoting complex binding	6	0.00658	0.986854
GOTERM_MF_DIRECT	GO:0003756~protein disulfide isomerase activity	9	0.006857	0.986854

GOTERM_MF_DIRECT	GO:1990459~transferrin receptor binding	7	0.009354	0.999504
GOTERM_MF_DIRECT	GO:0061630~ubiquitin protein ligase activity	68	0.010326	0.999504
GOTERM_MF_DIRECT	GO:0005154~epidermal growth factor receptor binding	12	0.013922	0.999504
GOTERM_MF_DIRECT	GO:0019899~enzyme binding	46	0.015252	0.999504
GOTERM_MF_DIRECT	GO:0051018~protein kinase A binding	9	0.017138	0.999504
GOTERM_MF_DIRECT	GO:0008017~microtubule binding	62	0.021024	0.999504
GOTERM_MF_DIRECT	GO:0045322~unmethylated CpG binding	5	0.022211	0.999504
GOTERM_MF_DIRECT	GO:0043531~ADP binding	10	0.02313	0.999504
GOTERM_MF_DIRECT	GO:0043425~bHLH transcription factor binding	9	0.025127	0.999504
GOTERM_MF_DIRECT	GO:0061629~RNA polymerase II sequence-specific DNA binding transcription factor binding	31	0.026026	0.999504
GOTERM_MF_DIRECT	GO:0004672~protein kinase activity	65	0.026405	0.999504
GOTERM_MF_DIRECT	GO:0001221~transcription cofactor binding	6	0.026768	0.999504
GOTERM_MF_DIRECT	GO:0004842~ubiquitin-protein transferase activity	41	0.026985	0.999504
GOTERM_MF_DIRECT	GO:0017116~single-stranded DNA-dependent ATP-dependent DNA helicase activity	7	0.027649	0.999504
GOTERM_MF_DIRECT	GO:0004861~cyclin-dependent protein serine/threonine kinase inhibitor activity	7	0.027649	0.999504
GOTERM_MF_DIRECT	GO:0003697~single-stranded DNA binding	27	0.032552	0.999504
GOTERM_MF_DIRECT	GO:0005547~phosphatidylinositol-3,4,5-trisphosphate binding	12	0.033084	0.999504
GOTERM_MF_DIRECT	GO:0047696~beta-adrenergic receptor kinase activity	4	0.033585	0.999504
GOTERM_MF_DIRECT	GO:0046923~ER retention sequence binding	4	0.033585	0.999504

GOTERM_MF_DIRECT	GO:0042393~histone binding	39	0.033862	0.999504
GOTERM_MF_DIRECT	GO:0042802~identical protein binding	236	0.036471	0.999504
GOTERM_MF_DIRECT	GO:0016922~ligand-dependent nuclear receptor binding	11	0.037542	0.999504
GOTERM_MF_DIRECT	GO:0035091~phosphatidylinositol binding	29	0.037987	0.999504
GOTERM_MF_DIRECT	GO:1990837~sequence-specific double-stranded DNA binding	36	0.038224	0.999504
GOTERM_MF_DIRECT	GO:0004674~protein serine/threonine kinase activity	76	0.038547	0.999504
GOTERM_MF_DIRECT	GO:0004703~G-protein coupled receptor kinase activity	5	0.043052	0.999504
GOTERM_MF_DIRECT	GO:0035174~histone serine kinase activity	5	0.043052	0.999504
GOTERM_MF_DIRECT	GO:0019208~phosphatase regulator activity	5	0.043052	0.999504
GOTERM_MF_DIRECT	GO:0001164~RNA polymerase I CORE element sequence-specific DNA binding	5	0.043052	0.999504
GOTERM_MF_DIRECT	GO:0002039~p53 binding	19	0.043161	0.999504
GOTERM_MF_DIRECT	GO:0030374~ligand-dependent nuclear receptor transcription coactivator activity	15	0.043292	0.999504
GOTERM_MF_DIRECT	GO:0043139~5'-3' DNA helicase activity	6	0.044181	0.999504
GOTERM_MF_DIRECT	GO:0019901~protein kinase binding	89	0.046037	0.999504
GOTERM_MF_DIRECT	GO:0019904~protein domain specific binding	40	0.046042	0.999504
GOTERM_MF_DIRECT	GO:0001664~G-protein coupled receptor binding	19	0.059312	0.999504
GOTERM_MF_DIRECT	GO:0015095~magnesium ion transmembrane transporter activity	7	0.060672	0.999504
GOTERM_MF_DIRECT	GO:0004707~MAP kinase activity	7	0.060672	0.999504
GOTERM_MF_DIRECT	GO:0008324~cation transmembrane transporter activity	7	0.060672	0.999504

GOTERM_MF_DIRECT	GO:0003684~damaged DNA binding	16	0.064953	0.999504
GOTERM_MF_DIRECT	GO:0004843~thiol-dependent ubiquitin-specific protease activity	28	0.069295	0.999504
GOTERM_MF_DIRECT	GO:0046975~histone methyltransferase activity (H3-K36 specific)	5	0.071666	0.999504
GOTERM_MF_DIRECT	GO:0016627~oxidoreductase activity, acting on the CH-CH group of donors	5	0.071666	0.999504
GOTERM_MF_DIRECT	GO:0005522~profilin binding	5	0.071666	0.999504
GOTERM_MF_DIRECT	GO:0003810~protein-glutamine gamma-glutamyltransferase activity	5	0.071666	0.999504
GOTERM_MF_DIRECT	GO:0032041~NAD-dependent histone deacetylase activity (H3-K14 specific)	5	0.071666	0.999504
GOTERM_MF_DIRECT	GO:0003678~DNA helicase activity	14	0.072411	0.999504
GOTERM_MF_DIRECT	GO:0016538~cyclin-dependent protein serine/threonine kinase regulator activity	12	0.080314	0.999504
GOTERM_MF_DIRECT	GO:0005248~voltage-gated sodium channel activity	9	0.081614	0.999504
GOTERM_MF_DIRECT	GO:0003688~DNA replication origin binding	9	0.081614	0.999504
GOTERM_MF_DIRECT	GO:0070577~lysine-acetylated histone binding	7	0.08326	0.999504
GOTERM_MF_DIRECT	GO:0022841~potassium ion leak channel activity	7	0.08326	0.999504
GOTERM_MF_DIRECT	GO:0071889~14-3-3 protein binding	7	0.08326	0.999504
GOTERM_MF_DIRECT	GO:0005102~receptor binding	56	0.088734	0.999504
GOTERM_MF_DIRECT	GO:0008022~protein C-terminus binding	33	0.089106	0.999504
GOTERM_MF_DIRECT	GO:0019003~GDP binding	18	0.090388	0.999504

GOTERM_MF_DIRECT	GO:0016491~oxidoreductase activity	46	0.092291	0.999504
GOTERM_MF_DIRECT	GO:0005243~gap junction channel activity	8	0.09454	0.999504
GOTERM_MF_DIRECT	GO:0005267~potassium channel activity	8	0.09454	0.999504
GOTERM_MF_DIRECT	GO:0016746~transferase activity, transferring acyl groups	12	0.096548	0.999504
GOTERM_MF_DIRECT	GO:0004519~endonuclease activity	13	0.099119	0.999504
