# Supplementary material for: Identification and functional prediction of long non-coding RNAs related to oxidative stress in the jejunum of piglets
Source: Anim Biosci. 2023 Aug 25;37(2):193–202. doi: 10.5713/ab.23.0202 (PMC10766486; doi:10.5713/ab.23.0202)
Supplement: Supplementary file 5 [file ab-23-0202-Supplementary-Table-5.pdf]

Supplementary Table 5. KEGG pathways of target genes of DE lncRNAs

Term	Count	PValue	FDR
ssc01100:Metabolic pathways	382	1.37E-06	3.29E-04
ssc04110:Cell cycle	48	3.88E-06	3.29E-04
ssc05207:Chemical carcinogenesis - receptor activation	69	4.53E-06	3.29E-04
ssc03430:Mismatch repair	15	5.11E-06	3.29E-04
ssc03030:DNA replication	20	8.61E-06	4.43E-04
ssc05213:Endometrial cancer	27	1.95E-05	8.36E-04
ssc04141:Protein processing in endoplasmic reticulum	56	7.17E-05	0.002633
ssc05208:Chemical carcinogenesis - reactive oxygen species	71	8.74E-05	0.002809
ssc05223:Non-small cell lung cancer	29	1.16E-04	0.003046
ssc04662:B cell receptor signaling pathway	31	1.19E-04	0.003046
ssc05210:Colorectal cancer	34	1.70E-04	0.003971
ssc04071:Sphingolipid signaling pathway	41	3.42E-04	0.006859
ssc05226:Gastric cancer	49	3.61E-04	0.006859
ssc05215:Prostate cancer	35	3.74E-04	0.006859
ssc04714:Thermogenesis	69	5.56E-04	0.009534
ssc00020:Citrate cycle	15	6.59E-04	0.010587
ssc04664:Fc epsilon RI signaling pathway	26	7.01E-04	0.010601
ssc04068:FoxO signaling pathway	43	7.67E-04	0.010955
ssc05212:Pancreatic cancer	28	8.60E-04	0.010976
ssc05230:Central carbon metabolism in cancer	26	9.07E-04	0.010976

ssc01522:Endocrine resistance	33	9.69E-04	0.010976
ssc05225:Hepatocellular carcinoma	51	9.70E-04	0.010976
ssc04722:Neurotrophin signaling pathway	40	0.001025	0.010976
ssc04114:Oocyte meiosis	40	0.001025	0.010976
ssc05166:Human T-cell leukemia virus 1 infection	66	0.001104	0.011351
ssc04218:Cellular senescence	48	0.001525	0.015074
ssc05200:Pathways in cancer	135	0.001731	0.016472
ssc05214:Glioma	26	0.001865	0.017121
ssc04910:Insulin signaling pathway	43	0.002064	0.018289
ssc04151:PI3K-Akt signaling pathway	93	0.002466	0.021125
ssc05216:Thyroid cancer	16	0.002739	0.021677
ssc04934:Cushing syndrome	46	0.002756	0.021677
ssc04152:AMPK signaling pathway	39	0.002783	0.021677
ssc03410:Base excision repair	15	0.003081	0.023291
ssc05415:Diabetic cardiomyopathy	59	0.003331	0.024266
ssc05218:Melanoma	25	0.003399	0.024266
ssc04015:Rap1 signaling pathway	61	0.003579	0.024861
ssc05224:Breast cancer	44	0.004269	0.028874
ssc04350:TGF-beta signaling pathway	31	0.005	0.032951
ssc04022:cGMP-PKG signaling pathway	49	0.005163	0.033171
ssc05220:Chronic myeloid leukemia	26	0.005346	0.033511
ssc04216:Ferroptosis	17	0.005788	0.034679
ssc04070:Phosphatidylinositol signaling system	31	0.005924	0.034679

ssc05202:Transcriptional misregulation in cancer	53	0.006052	0.034679
ssc04977:Vitamin digestion and absorption	12	0.006072	0.034679
ssc01200:Carbon metabolism	35	0.007008	0.039154
ssc04024:cAMP signaling pathway	61	0.007626	0.041701
ssc04510:Focal adhesion	56	0.00786	0.042086
ssc00983:Drug metabolism - other enzymes	23	0.008661	0.045425
ssc04010:MAPK signaling pathway	76	0.010186	0.052357
ssc04921:Oxytocin signaling pathway	43	0.010723	0.054034
ssc03460:Fanconi anemia pathway	18	0.012975	0.063141
ssc05231:Choline metabolism in cancer	30	0.013021	0.063141
ssc04928:Parathyroid hormone synthesis, secretion and action	32	0.014757	0.069755
ssc04922:Glucagon signaling pathway	31	0.014928	0.069755
ssc04014:Ras signaling pathway	63	0.015426	0.070793
ssc05012:Parkinson disease	70	0.015933	0.071838
ssc05010:Alzheimer disease	96	0.016522	0.07321
ssc05219:Bladder cancer	15	0.016912	0.073299
ssc00980:Metabolism of xenobiotics by cytochrome P450	20	0.017113	0.073299
ssc05221:Acute myeloid leukemia	22	0.017717	0.074643
ssc04072:Phospholipase D signaling pathway	42	0.019543	0.081009
ssc04972:Pancreatic secretion	30	0.019962	0.081433
ssc04330:Notch signaling pathway	20	0.020579	0.082129
ssc04917:Prolactin signaling pathway	23	0.021092	0.082129

ssc04520:Adherens junction	23	0.021092	0.082129
ssc04140:Autophagy - animal	40	0.023013	0.088274
ssc04914:Progesterone-mediated oocyte maturation	28	0.023574	0.089094
ssc04012:ErbB signaling pathway	26	0.024186	0.089427
ssc04724:Glutamatergic synapse	33	0.024426	0.089427
ssc04750:Inflammatory mediator regulation of TRP channels	32	0.024967	0.089427
ssc00040:Pentose and glucuronate interconversions	11	0.025053	0.089427
ssc05418:Fluid shear stress and atherosclerosis	39	0.026375	0.091839
ssc05165:Human papillomavirus infection	82	0.026444	0.091839
ssc04115:p53 signaling pathway	23	0.028834	0.098806
ssc04912:GnRH signaling pathway	27	0.031399	0.106169
ssc01524:Platinum drug resistance	23	0.033432	0.106169
ssc04270:Vascular smooth muscle contraction	38	0.033566	0.106169
ssc04919:Thyroid hormone signaling pathway	34	0.033825	0.106169
ssc04978:Mineral absorption	18	0.034176	0.106169
ssc04916:Melanogenesis	29	0.034202	0.106169
ssc00140:Steroid hormone biosynthesis	20	0.034244	0.106169
ssc05204:Chemical carcinogenesis - DNA adducts	19	0.034288	0.106169
ssc04390:Hippo signaling pathway	42	0.036208	0.110778
ssc04810:Regulation of actin cytoskeleton	56	0.038222	0.115524
ssc00053:Ascorbate and aldarate metabolism	10	0.03874	0.115524
ssc03420:Nucleotide excision repair	15	0.039526	0.115524

ssc04728:Dopaminergic synapse	36	0.039557	0.115524
ssc00240:Pyrimidine metabolism	18	0.040436	0.116764
ssc04611:Platelet activation	34	0.042033	0.120029
ssc04935:Growth hormone synthesis, secretion and action	33	0.043316	0.122333
ssc05205:Proteoglycans in cancer	53	0.045867	0.128128
ssc04210:Apoptosis	37	0.04686	0.129238
ssc04540:Gap junction	25	0.048191	0.129238
ssc04911:Insulin secretion	25	0.048191	0.129238
ssc04145:Phagosome	39	0.048276	0.129238
ssc05017:Spinocerebellar ataxia	39	0.052958	0.140311
ssc01210:2-Oxocarboxylic acid metabolism	8	0.054273	0.142329
ssc04971:Gastric acid secretion	22	0.058816	0.152684
ssc04530:Tight junction	43	0.059912	0.153975
ssc00830:Retinol metabolism	20	0.070278	0.178827
ssc00564:Glycerophospholipid metabolism	27	0.07189	0.181134
ssc04020:Calcium signaling pathway	60	0.073413	0.183066
ssc05016:Huntington disease	74	0.074081	0.183066
ssc03008:Ribosome biogenesis in eukaryotes	22	0.07527	0.183801
ssc04211:Longevity regulating pathway	25	0.076755	0.183801
ssc01230:Biosynthesis of amino acids	21	0.077509	0.183801
ssc00565:Ether lipid metabolism	15	0.07809	0.183801
ssc04976:Bile secretion	24	0.07929	0.183801
ssc04360:Axon guidance	46	0.07969	0.183801

ssc04062:Chemokine signaling pathway	46	0.07969	0.183801
ssc00512:Mucin type O-glycan biosynthesis	12	0.0801	0.183801
ssc04144:Endocytosis	59	0.081059	0.184356
ssc05222:Small cell lung cancer	26	0.082519	0.184412
ssc04666:Fc gamma R-mediated phagocytosis	26	0.082519	0.184412
ssc05022:Pathways of neurodegeneration - multiple diseases	109	0.089085	0.197369
ssc04660:T cell receptor signaling pathway	29	0.090344	0.198447
ssc03440:Homologous recombination	13	0.093958	0.201791
ssc00860:Porphyrin metabolism	12	0.095145	0.201791
ssc04150:mTOR signaling pathway	39	0.095345	0.201791
ssc00561:Glycerolipid metabolism	18	0.095726	0.201791
ssc00640:Propanoate metabolism	11	0.095792	0.201791
ssc04370:VEGF signaling pathway	17	0.098587	0.203848
ssc04932:Non-alcoholic fatty liver disease	40	0.099132	0.203848
ssc05161:Hepatitis B	42	0.099148	0.203848
